# The spoon illusion: A consistent rearward bias in human sound localisation

**DOI:** 10.1177/03010066251395028

**Published:** 2025-11-19

**Authors:** EunJi Baek, Min Hee Shim, Ecem Altan, Gene Tangtartharakul, Katherine Storrs, Paul Michael Corballis, Dietrich Samuel Schwarzkopf

**Affiliations:** 1 School of Optometry & Vision Science, University of Auckland, Auckland, New Zealand; 2 School of Psychology, University of Auckland, Auckland, New Zealand; 3 Experimental Psychology, University College London, London, United Kingdom

**Keywords:** audition, spatial cognition, 3D perception, illusion, spoons, weird

## Abstract

Most humans have only two ears. To know where a sound is in external space, our auditory system must therefore rely on the limited information received by these ears alone. In an adventurous late-night attempt to test blindfolded humans’ ability to achieve this feat, we discovered that we mishear the sound of two spoons being hit right in front of us as coming from behind us.

To test the consistency of this startling finding, we conducted a larger experiment on the senior author (DSS) and 11 naïve listeners (Figure 1A). We replicated a robust bias for listeners to hear sounds on the sagittal plane in front of them as coming from behind (Figure 1). No listener correctly localised sounds presented in front of their navel; in fact, only in a handful of trials did anyone report that sounds came from the front at all. Listeners were instead strongly biased to say frontal sounds came from behind or (somewhat less frequently) from above. They were better at locating sounds presented above the head, although they also often heard these as being behind the head. All listeners could tell with perfect accuracy whether sounds were on the left or right of the head.

**Figure 1.**
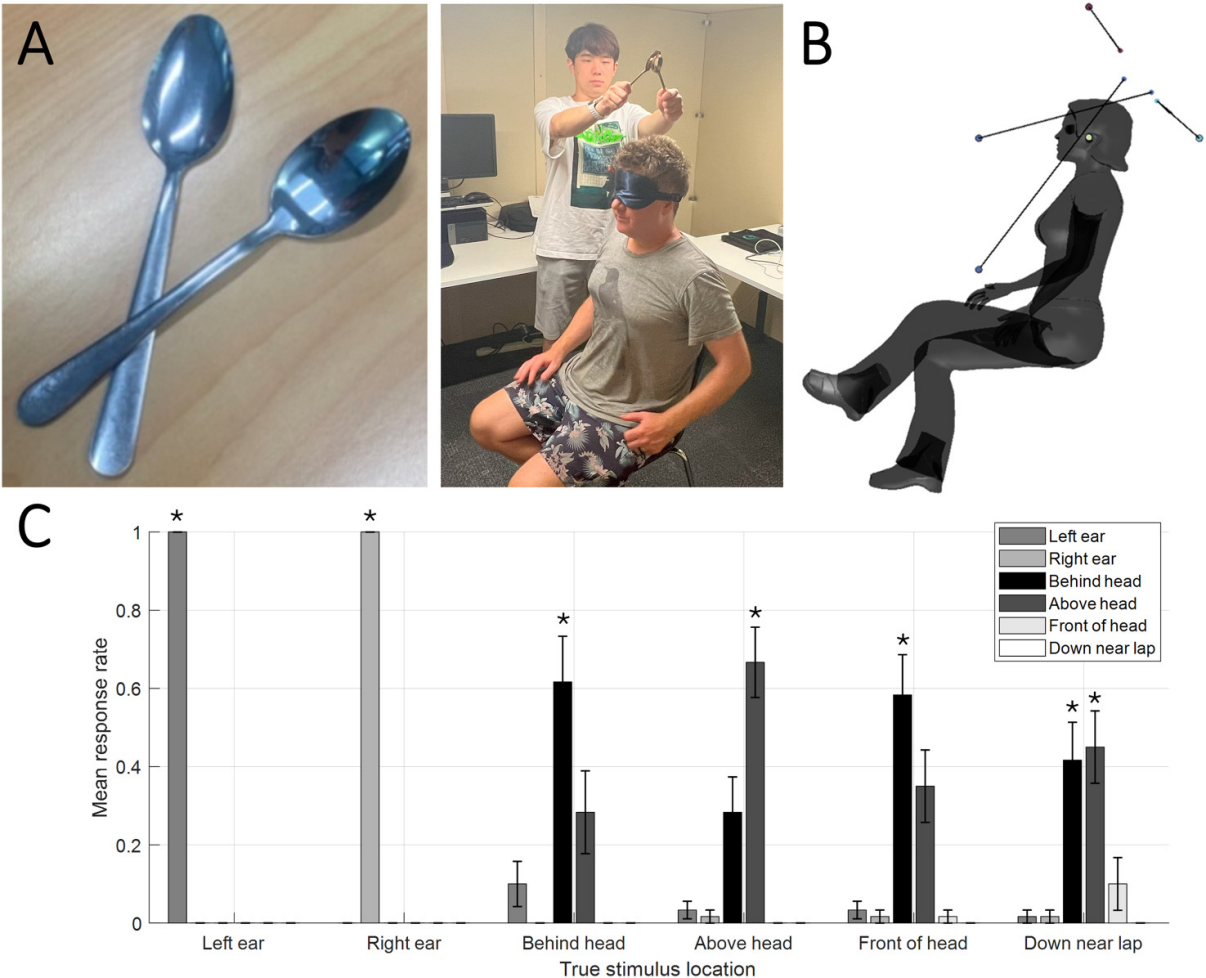
Rearward Bias Illusion. (A) Experimental design. The listener wore a blindfold and sat on a chair in the centre of the testing room. In each trial, one experimenter produced a sound by striking together two spoons approx. 15-20 cm from the listener's body in six different locations. Each location was presented five times per listener. The trial sequence was pseudorandomised via a custom-made MATLAB program running on a laptop. The listener verbally reported the perceived location of each sound, and another experimenter recorded this with a keypress. (B) Sagittal view of results. We tested six sound locations (indicated by the larger dots): (1) to the Left or (2) Right, (3) Behind, (4) Above, or (5) Behind the Head, or (6) Just Above the lap, approximately at the height of the navel, where we had first noticed the illusion. The solid lines to the smaller dots denote where participants responded on average that they had heard the sound. View an animation at: https://osf.io/prjcn (C) Bar chart showing responses per condition, averaged across listeners (error bars represent ±1 standard error of the mean). The x-axis indicates the true sound location stimulated. The colour scheme (see inset) denotes the proportion of trials listeners responded with a given location. Asterisks denote significance on one-tailed t-test vs. 1/6 at p < .05 (false-discovery rate corrected).

These results speak for themselves. However, in this digital age many scientists cannot believe results without inferential statistics. We therefore compared for each of the six stimulus locations whether listeners’ average response rate was greater than guessing (Figure 1C).

We have since learned of independent replications of this phenomenon by others and have also tentatively replicated it in other contexts. Our tests suggest that the bias persists in a sound-attenuated chamber and outdoors on a rural lawn >10 m from any walls or other structure, which suggests it is not due to sound reflections from the walls of the testing room. The illusion also occurs when listeners are standing upright, rather than sitting down, and with other sounds than hitting spoons. While these follow-up experiments all included non-naïve listeners and authors who had participated in the first experiment, we also included naïve listeners who nevertheless also experienced the illusion. We assert that the illusion is extremely compelling—even when one knows that some stimuli must come from the front, they sound distinctly as coming from behind. We encourage readers to attempt their own replication and report their results back to us. We note that the illusion makes for an excellent party trick.

What could explain this pronounced perceptual bias? Errors in sound localisation between frontal and rear locations typically occur when binaural cues, intensity and timing differences between the ears, are ambiguous (Fischer et al., 2020; Shinn-Cunningham et al., 2000; Wightman & Kistler, 1999). But crucially, the illusion we report goes far beyond front-back confusion: it is a pronounced directional *bias*, rather than merely a heightened error rate. Sounds in front of the listener are presumably louder than those coming from behind them and probably also differ in spectral characteristics (Blauert, 1996; Ege et al., 2018; Montagne & Zhou, 2018; Oldfield & Parker, 1984). However, it is unclear why this should result in compellingly hearing the sounds from one direction.

The illusion might reflect prior expectations that unseen sounds originate from outside the visual field. Although it is also unclear why this should happen during blindfolding when there is no external visual input at all. This hypothesis could be tested in listeners after extended visual deprivation and in congenitally blind listeners. Expectations could also be manipulated by pairing sounds with visual stimuli. Localisation performance is known to be affected by synchronous visual stimuli (Jack & Thurlow, 1973; Montagne & Zhou, 2018). Despite the simplicity of using only two spoons to create this illusion, future research should strive for more controlled conditions, using a multi-speaker setup that allows exact control over stimulus location, loudness, spectral attributes, and audiovisual manipulations.
